# Supplementary material for: No Evidence for Genetic Role of the Tumor Necrosis Factor Pathway in Parkinson’s Disease
Source: medRxiv. 2025 Sep 8:2025.09.06.25335210. Preprint. [Version 1] doi: 10.1101/2025.09.06.25335210 (PMC12440034; doi:10.1101/2025.09.06.25335210)
Supplement: Supplement 1 [file media-1.pdf]

Supplementary Table 1. Genes in the TNF signaling pathway and their functions

Gene	Full Gene Name	Function
TNF	Tumor Necrosis Factor	Encodes TNF-α, a pro-inflammatory cytokine mediating neuroinflammation.
TNFRSF1A	TNF Receptor Superfamily Member 1A	Encodes TNFRSF1A (TNFR1), the main receptor for TNF; triggers apoptosis or NF-κB-mediated survival/inflammatory signaling.
TNFRSF1B	TNF Receptor Superfamily Member 1B	Encodes TNFRSF1B (TNFR2), a TNF receptor mainly promoting cell survival and immune regulation via NF-κB activation.
TRADD	TNFRSF1A-associated via death domain	Encodes TRADD, an adaptor protein recruited by TNFR1; involved in NF-κB activation or, alternatively, apoptosis.
TRAF2	TNF Receptor Associated Factor 2	Encodes TRAF2, an E3 ubiquitin ligase recruited by TNFR1/TRADD, contributes to NF-κB activation.
TRAF5	TNF Receptor Associated Factor 5	Encodes TRAF5, contributes to NF-κB signaling.
TRAF1	TNF Receptor Associated Factor 1	Encodes TRAF1, which together with TRAF2 contributes to NF-κB activation.
CASP8	Caspase 8	Encodes CASP8, involved in NF-κB activation or, alternatively, apoptosis.
NFKB1 & *NFKB2*	Nuclear Factor Kappa B Subunit 1 and 2	Encode NF-κB subunits, which contributes to the prevention of TNF-induced apoptosis through the expression of anti-apoptotic and inflammatory genes such as *TRAF1*, *TRAF2*, and *CASP8*.

Supplementary Table 2. Summary-data-based Mendelian Randomization studies between TNF-related genes expression loci and Parkinson's disease risk and progression, and REM sleep behavior disorder risk

Gene	topSNP	b.SMR	se.SMR	p.SMR	pFDR.SMR	p.multi SMR	pFDR.multi SMR	p.HEIDI	eQTL	Tissue	Disease
CASP8	rs12990906	0.119664	0.131149	0.362	0.684	0.033	0.607	0.406	BrainMeta	Brain Cortex	RBDrisk
NFKB1	rs230541	0.145106	0.155188	0.35	0.684	0.35	0.752	0.495	BrainMeta	Brain Cortex	RBDrisk
TNFRSF1A	rs4149577	0.0886967	0.252064	0.725	0.914	0.725	0.952	0.085	BrainMeta	Brain Cortex	RBDrisk
TRAF1	rs4310279	0.022075	0.0979041	0.822	0.914	0.976	0.976	0.931	BrainMeta	Brain Cortex	RBDrisk
TRAF5	rs2175633	0.187063	0.147401	0.204	0.623	0.204	0.701	0.351	BrainMeta	Brain Cortex	RBDrisk
CASP8	rs2540334	-0.448773	0.403737	0.266	0.678	0.515	0.874	0.255	BrainMeta	Brain Cortex	moca
NFKB1	rs3774964	0.662164	0.408089	0.105	0.623	0.105	0.607	0.719	BrainMeta	Brain Cortex	moca
TRAF1	rs6478485	-0.293944	0.312833	0.347	0.684	0.516	0.874	0.928	BrainMeta	Brain Cortex	moca
TRAF5	rs12095062	-0.515413	0.646322	0.425	0.765	0.425	0.792	0.093	BrainMeta	Brain Cortex	moca
CASP8	rs2540334	0.185759	0.0840138	0.027	0.623	0.193	0.701	0.668	BrainMeta	Brain Cortex	updrs3
NFKB1	rs3774964	0.0239799	0.0802935	0.765	0.914	0.765	0.952	0.405	BrainMeta	Brain Cortex	updrs3
NFKB2	rs36226954	0.280081	0.358733	0.435	0.765	0.435	0.792	0.141	BrainMeta	Brain Cortex	updrs3
TRAF1	rs6478485	-0.00678228	0.0628061	0.914	0.914	0.531	0.874	0.422	BrainMeta	Brain Cortex	updrs3
TRAF5	rs9887769	0.289495	0.149409	0.053	0.623	0.053	0.607	0.146	BrainMeta	Brain Cortex	updrs3
CASP8	rs12990906	0.163257	0.145745	0.263	0.678	0.686	0.946	0.764	BrainMeta	Brain Cortex	motorPD
NFKB1	rs230541	0.213339	0.17085	0.212	0.623	0.212	0.701	0.338	BrainMeta	Brain Cortex	motorPD
TNFRSF1A	rs4149577	0.134531	0.278831	0.629	0.871	0.629	0.917	NA	BrainMeta	Brain Cortex	motorPD
TRAF1	rs4310279	-0.128322	0.103322	0.214	0.623	0.629	0.917	0.363	BrainMeta	Brain Cortex	motorPD
TRAF5	rs9887769	0.148638	0.160486	0.354	0.684	0.354	0.752	0.612	BrainMeta	Brain Cortex	motorPD
CASP8	rs2110690	0.109681	0.0828049	0.185	0.623	0.139	0.644	0.466	GTEx	Brain Cortex	RBDrisk
CASP8	rs2110690	-0.274977	0.204904	0.18	0.623	0.002	0.102	0.236	GTEx	Whole Blood	RBDrisk
TRAF1	rs4310279	0.0347127	0.154016	0.822	0.914	0.822	0.952	0.793	GTEx	Brain Caudate basal ganglia	RBDrisk
TRAF1	rs4310279	0.0338337	0.15011	0.822	0.914	0.95	0.969	0.971	GTEx	Brain Cortex	RBDrisk
TRAF1	rs6478485	-0.0914507	0.417746	0.827	0.914	0.599	0.917	0.593	GTEx	Whole Blood	RBDrisk
TRAF2	rs7854924	0.729355	0.468016	0.119	0.623	0.119	0.607	0.428	GTEx	Whole Blood	RBDrisk
CASP8	rs2110690	0.120428	0.203642	0.554	0.853	0.821	0.952	0.379	GTEx	Brain Cortex	moca
CASP8	rs2110690	-0.30192	0.50923	0.553	0.853	0.647	0.917	0.821	GTEx	Whole Blood	moca
TRAF1	rs6478485	-0.428312	0.45943	0.351	0.684	0.351	0.752	NA	GTEx	Brain Cortex	moca
TRAF1	rs6478485	-0.999101	1.07165	0.351	0.684	0.351	0.752	NA	GTEx	Whole Blood	moca
CASP8	rs2110690	0.0780834	0.0424785	0.066	0.623	0.094	0.607	0.937	GTEx	Brain Cortex	updrs3
CASP8	rs2110690	-0.19576	0.10382	0.059	0.623	0.284	0.752	0.93	GTEx	Whole Blood	updrs3

TRAF1	rs6478485	-0.00988261	0.0915258	0.914	0.914	0.914	0.952	NA	GTEx	Brain Cortex	updrs3
TRAF1	rs6478485	-0.0230526	0.213497	0.914	0.914	0.914	0.952	NA	GTEx	Whole Blood	updrs3
CASP8	rs2110690	0.0443771	0.0926974	0.632	0.871	0.915	0.952	0.983	GTEx	Brain Cortex	motorPD
CASP8	rs2110690	-0.111256	0.232006	0.632	0.871	0.806	0.952	0.826	GTEx	Whole Blood	motorPD
TRAF1	rs4310279	-0.201785	0.164471	0.22	0.623	0.22	0.701	0.189	GTEx	Brain Caudate basal ganglia	motorPD
TRAF1	rs4310279	-0.196676	0.160129	0.219	0.623	0.392	0.792	0.737	GTEx	Brain Cortex	motorPD
TRAF1	rs6478485	-0.263261	0.449534	0.558	0.853	0.825	0.952	0.575	GTEx	Whole Blood	motorPD
TRAF2	rs7854924	0.500918	0.478137	0.295	0.684	0.295	0.752	NA	GTEx	Whole Blood	motorPD
CASP8	rs12990906	0.0245294	0.0187634	0.191	0.623	0.565	0.9	0.679	BrainMeta	Brain Cortex	PDrisk
NFKB1	rs230541	-0.0372947	0.0222532	0.094	0.623	0.094	0.607	0.353	BrainMeta	Brain Cortex	PDrisk
NFKB2	rs36226954	0.0507343	0.0317294	0.11	0.623	0.11	0.607	0.121	BrainMeta	Brain Cortex	PDrisk
TNFRSF1A	rs4149577	0.0127618	0.0376632	0.735	0.914	0.735	0.952	0.756	BrainMeta	Brain Cortex	PDrisk
TRAF1	rs4310279	0.00206971	0.0129281	0.873	0.914	0.071	0.607	0.364	BrainMeta	Brain Cortex	PDrisk
TRAF5	rs2175633	0.040204	0.0203418	0.048	0.623	0.048	0.607	0.419	BrainMeta	Brain Cortex	PDrisk
CASP8	rs2110690	0.00643638	0.0113066	0.569	0.853	0.353	0.752	0.398	GTEx	Brain Cortex	PDrisk
CASP8	rs2110690	-0.0161364	0.0282788	0.568	0.853	0.185	0.701	0.152	GTEx	Whole Blood	PDrisk
TRAF1	rs4310279	0.0032546	0.0203335	0.873	0.914	0.873	0.952	0.781	GTEx	Brain Caudate basal ganglia	PDrisk
TRAF1	rs4310279	0.00317219	0.0198182	0.873	0.914	0.421	0.792	0.554	GTEx	Brain Cortex	PDrisk
TRAF1	rs6478485	-0.0774568	0.0596796	0.194	0.623	0.267	0.752	0.905	GTEx	Whole Blood	PDrisk
TRAF2	rs7854924	-0.0089741	0.061741	0.884	0.914	0.884	0.952	0.626	GTEx	Whole Blood	PDrisk

SNP – Single Nucleotide Polymorphism; b – effect size (regression coefficient); SMR – Summary-data-based Mendelian Randomization; SE – Standard error; FDR – false discovery rate; eQTL – expression quantitative trait loci.

Supplementary Table 3. Rare variant association analysis in TNF-related genes in Parkinson's disease cases and controls

SetID	p.value	p.FDR	N.Marker
TNFRSF1B_ALL_UKBPD	0.104	0.598	3329
TRAF5_ALL_UKBPD	0.447	0.855	3475
CASP8_ALL_UKBPD	1	1	1930
NFKB1_ALL_UKBPD	0.21	0.823	9006
TNF_ALL_UKBPD	0.757	0.873	186
TRAF1_ALL_UKBPD	0.746	0.873	1873
TRAF2_ALL_UKBPD	0.644	0.855	4444
NFKB2_ALL_UKBPD	0.672	0.866	511
TNFRSF1A_ALL_UKBPD	0.719	0.873	1036
TRADD_ALL_UKBPD	0.349	0.855	420
TNFRSF1B_CADD_UKBPD	0.613	0.855	8
TRAF5_CADD_UKBPD	0.031	0.598	42
CASP8_CADD_UKBPD	0.103	0.598	8
NFKB1_CADD_UKBPD	0.219	0.826	50
TNF_CADD_UKBPD	0.836	0.902	6
TRAF1_CADD_UKBPD	0.74	0.873	24
TRAF2_CADD_UKBPD	0.501	0.855	37
NFKB2_CADD_UKBPD	0.625	0.855	31
TNFRSF1A_CADD_UKBPD	1	1	9
TRADD_CADD_UKBPD	0.853	0.902	3
TNFRSF1B_LOF_UKBPD	0.62	0.855	3
TRAF5_LOF_UKBPD	0.681	0.866	14
CASP8_LOF_UKBPD	0.302	0.855	6
NFKB1_LOF_UKBPD	0.507	0.855	2
TNF_LOF_UKBPD	0.502	0.855	1
TRAF1_LOF_UKBPD	0.707	0.873	3
TRAF2_LOF_UKBPD	0.094	0.598	4
NFKB2_LOF_UKBPD	0.39	0.855	1
TNFRSF1A_LOF_UKBPD	0.537	0.855	2
TNFRSF1B_MISSENSE_UKBPD	0.559	0.855	71
TRAF5_MISSENSE_UKBPD	0.075	0.598	75
CASP8_MISSENSE_UKBPD	0.248	0.855	30
NFKB1_MISSENSE_UKBPD	0.2	0.823	148
TNF_MISSENSE_UKBPD	0.631	0.855	23
TRAF1_MISSENSE_UKBPD	0.561	0.855	69
TRAF2_MISSENSE_UKBPD	0.514	0.855	87
NFKB2_MISSENSE_UKBPD	0.588	0.855	13
TNFRSF1A_MISSENSE_UKBPD	0.421	0.855	50
TRADD_MISSENSE_UKBPD	0.362	0.855	43
TNFRSF1B_ALL_AMPPD	0.154	0.756	236

TRAF5_ALL_AMPPD	0.01	0.327	169
CASP8_ALL_AMPPD	0.307	0.855	86
NFKB1_ALL_AMPPD	0.293	0.855	474
TNF_ALL_AMPPD	0.181	0.808	17
TRAF1_ALL_AMPPD	0.557	0.855	137
TRAF2_ALL_AMPPD	0.376	0.855	252
NFKB2_ALL_AMPPD	0.282	0.855	50
TNFRSF1A_ALL_AMPPD	0.393	0.855	106
TRADD_ALL_AMPPD	0.773	0.875	27
TRAF5_LOF_AMPPD	0.617	0.855	1
TNFRSF1B_MISSENSE_AMPPD	0.097	0.598	12
TRAF5_MISSENSE_AMPPD	0.353	0.855	13
CASP8_MISSENSE_AMPPD	0.542	0.855	4
NFKB1_MISSENSE_AMPPD	0.73	0.873	18
TNF_MISSENSE_AMPPD	0.052	0.598	4
TRAF1_MISSENSE_AMPPD	0.006	0.312	17
TRAF2_MISSENSE_AMPPD	0.697	0.873	10
NFKB2_MISSENSE_AMPPD	0.878	0.905	2
TNFRSF1A_MISSENSE_AMPPD	0.053	0.598	12
TRADD_MISSENSE_AMPPD	0.499	0.855	2
TNFRSF1B_CADD_AMPPD	0.203	0.823	2
TRAF5_CADD_AMPPD	0.551	0.855	6
CASP8_CADD_AMPPD	0.777	0.875	2
NFKB1_CADD_AMPPD	0.102	0.598	9
TNF_CADD_AMPPD	0.281	0.855	2
TRAF1_CADD_AMPPD	0.057	0.598	10
TRAF2_CADD_AMPPD	0.578	0.855	6
NFKB2_CADD_AMPPD	0.275	0.855	8
TNFRSF1A_CADD_AMPPD	0.07	0.598	5
CASP8_ALL_META	1	1	2016
CASP8_CADD_META	0.114	0.621	10
CASP8_MISSENSE_META	0.37	0.855	34
NFKB1_CADD_META	0.645	0.855	59
NFKB1_MISSENSE_META	0.31	0.855	166
NFKB2_ALL_META	0.811	0.893	561
NFKB2_CADD_META	0.757	0.873	39
NFKB2_MISSENSE_META	0.865	0.902	15
TNF_ALL_META	0.665	0.866	203
TNF_CADD_META	0.864	0.902	8
TNF_MISSENSE_META	0.079	0.598	27
TNFRSF1A_ALL_META	0.371	0.855	1142
TNFRSF1A_CADD_META	0.513	0.855	14
TNFRSF1A_MISSENSE_META	0.523	0.855	62

TNFRSF1B_ALL_META	0.067	0.598	3565
TNFRSF1B_CADD_META	0.506	0.855	10
TNFRSF1B_MISSENSE_META	0.395	0.855	83
TRADD_ALL_META	0.633	0.855	447
TRADD_MISSENSE_META	0.5	0.855	45
TRAF1_ALL_META	0.854	0.902	2010
TRAF1_CADD_META	0.181	0.808	34
TRAF1_MISSENSE_META	0.228	0.826	86
TRAF2_ALL_META	0.533	0.855	4696
TRAF2_CADD_META	0.381	0.855	43
TRAF2_MISSENSE_META	0.404	0.855	97
TRAF5_ALL_META	0.128	0.662	3644
TRAF5_CADD_META	0.005	0.312	48
TRAF5_LOF_META	0.79	0.88	15
TRAF5_MISSENSE_META	0.037	0.598	88
PATHWAY_CADD_UKBPD	0.693	0.721	218
PATHWAY_LOF_UKBPD	0.484	0.721	36
PATHWAY_MISSENSE_UKBPD	0.149	0.346	609
PATHWAY_CADD_AMPPD	0.068	0.346	50
PATHWAY_LOF_AMPPD	0.617	0.721	1
PATHWAY_MISSENSE_AMPPD	0.154	0.346	94
PATHWAY_CADD_META	0.721	0.721	268
PATHWAY_LOF_META	0.661	0.721	37
PATHWAY_MISSENSE_META	0.113	0.346	703

FDR – false discovery rate; UKBPD – UK Biobank including Parkinson's disease cases and controls; AMPPD – Accelerating Medicines Partnership Parkinson’s Disease; CADD – Combined Annotation Dependent Depletion (variants with score >20); LOF – loss-of-function; META – meta-analysis of the cohorts.

Supplementary Table 4. Rare variant association analysis in TNF-related genes in Parkinson's disease cases, proxy cases, and controls

SetID	p.value	p.FDR	N.Marker
TNFRSF1B_ALL_AMPPD	0.154	0.71	236
TRAF5_ALL_AMPPD	0.01	0.245	169
CASP8_ALL_AMPPD	0.307	0.753	86
NFKB1_ALL_AMPPD	0.293	0.753	474
TNF_ALL_AMPPD	0.181	0.71	17
TRAF1_ALL_AMPPD	0.557	0.805	137
TRAF2_ALL_AMPPD	0.376	0.77	252
NFKB2_ALL_AMPPD	0.282	0.753	50
TNFRSF1A_ALL_AMPPD	0.393	0.77	106
TRADD_ALL_AMPPD	0.773	0.856	27
TRAF5_LOF_AMPPD	0.617	0.805	1
TNFRSF1B_MISSENSE_AMPPD	0.097	0.555	12
TRAF5_MISSENSE_AMPPD	0.353	0.77	13
CASP8_MISSENSE_AMPPD	0.542	0.805	4
NFKB1_MISSENSE_AMPPD	0.73	0.822	18
TNF_MISSENSE_AMPPD	0.052	0.418	4
TRAF1_MISSENSE_AMPPD	0.006	0.228	17
TRAF2_MISSENSE_AMPPD	0.697	0.805	10
NFKB2_MISSENSE_AMPPD	0.878	0.935	2
TNFRSF1A_MISSENSE_AMPPD	0.053	0.418	12
TRADD_MISSENSE_AMPPD	0.499	0.805	2
TNFRSF1B_CADD_AMPPD	0.203	0.753	2
TRAF5_CADD_AMPPD	0.551	0.805	6
CASP8_CADD_AMPPD	0.777	0.856	2
NFKB1_CADD_AMPPD	0.102	0.555	9
TNF_CADD_AMPPD	0.281	0.753	2
TRAF1_CADD_AMPPD	0.057	0.418	10
TRAF2_CADD_AMPPD	0.578	0.805	6
NFKB2_CADD_AMPPD	0.275	0.753	8
TNFRSF1A_CADD_AMPPD	0.07	0.456	5
CASP8_ALL_META	0.007	0.228	2214
TNF_ALL_META	0.544	0.805	221
TNFRSF1B_ALL_META	0.811	0.883	3899
TRAF5_ALL_META	0.18	0.71	3990
CASP8_CADD_META	0.652	0.805	12
CASP8_MISSENSE_META	0.715	0.815	38
NFKB1_CADD_META	0.598	0.805	62
NFKB1_MISSENSE_META	0.457	0.804	180
NFKB2_ALL_META	0.295	0.753	605
NFKB2_CADD_META	0.167	0.71	41

NFKB2_MISSENSE_META	0.296	0.753	19
TNF_CADD_META	1	1	10
TNF_MISSENSE_META	0.347	0.77	35
TNFRSF1A_ALL_META	0.887	0.935	1262
TNFRSF1A_CADD_META	0.35	0.77	14
TNFRSF1A_MISSENSE_META	0.665	0.805	68
TNFRSF1B_CADD_META	0.672	0.805	12
TNFRSF1B_MISSENSE_META	0.852	0.918	93
TRADD_ALL_META	0.372	0.77	499
TRADD_MISSENSE_META	0.476	0.804	47
TRAF1_ALL_META	0.29	0.753	2204
TRAF1_CADD_META	0.175	0.71	35
TRAF1_MISSENSE_META	0.252	0.753	90
TRAF2_ALL_META	0.433	0.785	5111
TRAF2_CADD_META	0.174	0.71	47
TRAF2_MISSENSE_META	0.034	0.418	105
TRAF5_CADD_META	0.032	0.418	51
TRAF5_LOF_META	0.356	0.77	17
TRAF5_MISSENSE_META	0.469	0.804	97
TNFRSF1B_ALL_UKBPPROXY	0.937	0.977	3663
TRAF5_ALL_UKBPPROXY	0.564	0.805	3821
CASP8_ALL_UKBPPROXY	0.007	0.228	2128
TNF_ALL_UKBPPROXY	0.504	0.805	204
TRAF1_ALL_UKBPPROXY	0.06	0.418	2067
TRAF2_ALL_UKBPPROXY	0.272	0.753	4859
NFKB2_ALL_UKBPPROXY	0.255	0.753	555
TNFRSF1A_ALL_UKBPPROXY	1	1	1156
TRADD_ALL_UKBPPROXY	0.345	0.77	472
TNFRSF1B_CADD_UKBPPROXY	0.459	0.804	10
TRAF5_CADD_UKBPPROXY	0.045	0.418	45
CASP8_CADD_UKBPPROXY	0.65	0.805	10
NFKB1_CADD_UKBPPROXY	0.413	0.779	53
TNF_CADD_UKBPPROXY	1	1	8
TRAF1_CADD_UKBPPROXY	0.385	0.77	25
TRAF2_CADD_UKBPPROXY	0.232	0.753	41
NFKB2_CADD_UKBPPROXY	0.086	0.527	33
TNFRSF1A_CADD_UKBPPROXY	0.66	0.805	9
TRADD_CADD_UKBPPROXY	0.386	0.77	3
TNFRSF1B_LOF_UKBPPROXY	0.653	0.805	4
TRAF5_LOF_UKBPPROXY	0.305	0.753	16
CASP8_LOF_UKBPPROXY	0.639	0.805	8
NFKB1_LOF_UKBPPROXY	0.057	0.418	4
TNF_LOF_UKBPPROXY	0.567	0.805	1

TRAF1_LOF_UKBPPROXY	0.401	0.771	3
TRAF2_LOF_UKBPPROXY	0.026	0.418	7
NFKB2_LOF_UKBPPROXY	1	1	1
TNFRSF1A_LOF_UKBPPROXY	0.647	0.805	3
TRADD_LOF_UKBPPROXY	0.136	0.704	1
TNFRSF1B_MISSENSE_UKBPPROXY	0.601	0.805	81
TRAF5_MISSENSE_UKBPPROXY	0.555	0.805	84
CASP8_MISSENSE_UKBPPROXY	0.627	0.805	34
NFKB1_MISSENSE_UKBPPROXY	0.424	0.784	162
TNF_MISSENSE_UKBPPROXY	0.698	0.805	31
TRAF1_MISSENSE_UKBPPROXY	0.677	0.805	73
TRAF2_MISSENSE_UKBPPROXY	0.045	0.418	95
NFKB2_MISSENSE_UKBPPROXY	0.224	0.753	17
TNFRSF1A_MISSENSE_UKBPPROXY	0.682	0.805	56
TRADD_MISSENSE_UKBPPROXY	0.566	0.805	45
PATHWAY_MISSENSE_UKBPPROXY	0.791	0.791	678
PATHWAY_LOF_UKBPPROXY	0.198	0.338	48
PATHWAY_CADD_UKBPPROXY	0.117	0.338	237
PATHWAY_CADD_AMPPD	0.068	0.338	50
PATHWAY_LOF_AMPPD	0.617	0.791	1
PATHWAY_MISSENSE_AMPPD	0.154	0.338	94
PATHWAY_CADD_META	0.159	0.338	287
PATHWAY_LOF_META	0.225	0.338	49
PATHWAY_MISSENSE_META	0.723	0.791	772

FDR – false discovery rate; UKBPROXY – UK Biobank including Parkinson’s disease cases, proxy cases, and controls; AMPPD – Accelerating Medicines Partnership Parkinson’s Disease; CADD – Combined Annotation Dependent Depletion (variants with score >20); LOF – loss-of-function; META – meta-analysis of the cohorts.

Supplementary Table 5. Study population for pathway specific polygenic risk score analysis of TNF-related genes

Cohort	N_controls	N_cases	N_Male	N_Female	Mean_age
McGill	2127	3242	3098	2271	55.88
PPMI	164	417	387	194	60.18
APDGC	302	621	599	324	78.89
IPDGC	5480	5229	6421	4288	62.78
NINDS	790	896	865	821	62.64
NGRC	1968	1972	2092	1848	64.29
UKB	65720	3286	33999	35007	63.84

PPMI- Parkinson's Progression Markers Initiative; APDGC- Autopsy-Confirmed Parkinson Disease GWAS Consortium; IPDGC -International Parkinson Disease Genomics Consortium; NINDS- National Institute of Neurological Disorders and Stroke Repository Parkinson's Disease Collection; NGRC- NeuroGenetics Research Consortium; UKB-UK Biobank

Supplementary Table 6. Pathway specific polygenic risk score analysis of TNF-related genes in Parkinson's disease

Cohort	P	OR	SE	95%CI
McGill	0.000491	0.895308	0.028407352	[0.8413; 0.9528]
PPMI	0.500551	0.93519	0.093023952	[0.7695; 1.1365]
APDGC	0.957311	0.996073	0.07322568	[0.8624; 1.1504]
IPDGC	0.468675	1.014351	0.019945826	[0.9760; 1.0542]
NINDS	0.110751	1.086555	0.056556286	[0.9812; 1.2033]
NGRC	0.592286	0.980538	0.035986092	[0.9125; 1.0537]
UKB	0.046068	1.039793	0.020340316	[1.0007; 1.0804]
Random effects model	0.8404	0.9951		[0.9482; 1.0442]

PPMI- Parkinson's Progression Markers Initiative; APDGC- Autopsy-Confirmed Parkinson Disease GWAS Consortium; IPDGC -International Parkinson Disease Genomics Consortium; NINDS- National Institute of Neurological Disorders and Stroke Repository Parkinson's Disease Collection; NGRC- NeuroGenetics Research Consortium; UKB-UK Biobank; OR- odds ratio; SE-standard error; CI- confidence interval
